# Effects of estradiol and FSH on leptin levels in women with suppressed pituitary

**DOI:** 10.1186/1477-7827-10-45

**Published:** 2012-06-15

**Authors:** Selmo Geber, Augusto HF Brandão, Marcos Sampaio

**Affiliations:** 1 Origen, Center for Reproductive Medicine, Avenida do Contorno 7747, Lourdes, CEP, 30110120, Belo Horizonte, MG, Brazil; 2 Medical School of the Universidade Federal de Minas Gerais, Belo Horizonte, Brazil

**Keywords:** Leptin, Estradiol, Follicle stimulating hormone

## Abstract

**Background:**

Female fertility depends on adequate nutrition and energy reserves, suggesting a correlation between the metabolic reserve and reproductive capacity. Leptin regulates body weight and energy homeostasis. The aim of this study was to investigate whether estradiol or FSH alone has a direct effect on the production of leptin.

**Methods:**

A total of 64 patients submitted to controlled ovarian hyperstimulation with recombinant FSH for assisted reproduction and 20 patients using estradiol valerate for endometrial preparation for oocyte donation treatment were included in the study. All patients used GnRH analogues before starting treatment to achieve pituitary suppression. Blood samples for hormonal measurements were collected before starting and after completing the respective treatments. Data were analyzed statistically by the chi-square test, Student’s *t*-test and Pearson’s correlation test.

**Results:**

We observed an elevation of serum leptin levels secondary to the increase in estradiol, in the absence of influence of any other ovarian or pituitary hormone. The rising rate of leptin levels was higher in women treated with recombinant FSH, which also had higher levels of estradiol, than in those treated with estradiol valerate.

**Conclusions:**

This study demonstrates a correlation between serum levels of estradiol and leptin, suggesting that estradiol is an important regulator of leptin production and that its effects can be amplified by its association with FSH.

## Background

The relationship between body fat and the reproductive process has been studied for many years. Fertility depends on a correct nutrition and energy reserves, what creates a correlation between the metabolic reserve and reproductive capacity [1]. Women with extreme dietetic restrictions and those who are overweight show alterations in the reproductive axis. Obese women also have an early menarche than those with normal weight. This can be explained by the fact that menstrual cycle initiates only when the body weight reaches a critical mass, around 48 Kg or contains approximately 22% of body fat and obese women enter this critical weight range at a younger age [2]. Irregular menstrual cycles are frequent among women too thin, those with eating disorders such as bulimia or nervous anorexia and athletes submitted to long-term exercises [3].

Leptin is a protein codified by the obesity gene (ob), secreted by the adipocytes [4], which acts in the Central Nervous System as a signal to regulate body weight and energy homeostasis [5,6]. Initially, leptin was considered an anti-obesity hormone [7], but experimental evidences have shown that this molecule also participates in many metabolic and endocrine processes, including the reproductive function [8,9]. The presence of leptin receptors at all levels of the hypothalamic-pituitary-ovarian axis indicates a correlation between nutrition and reproduction, possibly through complex paracrine and/or endocrine interactions [10].

Although leptin receptors have been demonstrated in human ovaries, there is no information concerning leptin effects on follicular cells [11]. Contradictory results have been reported concerning the effects of estradiol and follicle-stimulating hormone (FSH) on leptin production by adipocytes [12-15]. Therefore the aim of this study was to investigate whether estradiol or FSH has a direct effect on the production of leptin.

## Methods

### Patients

This prospective study included a total of 84 patients who underwent infertility treatment through assisted reproduction technique at ORIGEN, Centre of Reproductive Medicine, Belo Horizonte, Brazil. This study was approved by our local ethics committee, according to Brazilian ethics and legal regulation, and all patients signed an Informed Consent Form. Patients with tubal or male factor infertility who had serum FSH concentrations <15 IU/l on day 3 of the menstrual cycle were submitted to controlled ovarian superovulation for IVF/ICSI (GnRHa/FSH Group) (n = 64). Patients with poor ovarian reserve, i.e., with serum FSH concentrations >15 IU/l and serum estradiol levels <60 pg/ml on day 3 of the menstrual cycle were submitted do oocyte donation (OD) treatment (GnRHa/E_2_ Group) (n = 20). Patients with endocrine disorders were not included. Body Mass Index (BMI) was calculated using the patient’s body weight (Kg) divided by the square of their height (meters). Women were considered obese when BMI was >30 kg/m^2^. All patients had normal glucose levels.

### Controlled ovarian stimulation

All patients of group 1 (GnRHa/FSH) submitted to IVF/ICSI had the same long protocol for controlled ovarian stimulation using the same hormones and the same criteria for dose tailoring. Treatment started with subcutaneous administration of 3.6 mg of GnRHa (Goserelin, Zoladex; Zeneca, Brazil) for suppression of pituitary function on day 2 of the menstrual cycle. To confirm pituitary down-regulation, serum estradiol (E_2_) levels and vaginal ultrasound were performed ~ 10 days later. If the estradiol concentration was <30 pg/ml and the ultrasound showed an endometrial thickness of <3 mm, pituitary suppression was confirmed. Otherwise, serum estradiol and vaginal ultrasound were repeated every other day until suppression was achieved [16].

After confirmation of suppression, patients were superovulated with daily subcutaneous injections of recombinant FSH (rFSH – GonalF – Serono – Brazil). The starting dose of rFSH was defined according to patient’s age, i.e., 225 IU for <35 years old, 300 IU for 35 to 37 years old and 375 IU for > 37 years old. The dose was adjusted according to the ovarian response measured by E_2_ levels and follicular growth monitored by vaginal ultrasound (Tosbee- Toshiba- Japan). Recombinant hCG (rhCG- 250 mcg – Ovidrel – Serono – Brazil) was given when at least two follicles reached a mean size of 17 mm with corresponding E_2_ levels (~ 200 pg/mL per follicle) [17]. Oocyte retrieval was performed ~34 hours after rhCG injection by vaginal ultrasound guided aspiration.

### Endometrial preparation

All patients in group 2 (GnRHa/E_2_) submitted to oocyte donation had the same endometrial preparation. Treatment started with pituitary suppression, as described for patients in group 1 (GnRHa/FSH). After pituitary down-regulation was confirmed, estradiol valerate (E_2_V – Primogyna – Bayer – Brazil) was given to the patients for endometrial preparation. Patients started with 2 mg/day from day 1 – 5, then the dose was increased to 4 mg/day from day 6 – 10, and increased to 6 mg/day from day 11 until 12 weeks of pregnancy. After 15 days of treatment, endometrial preparation was confirmed if E_2_ levels were >250 pg/ml and vaginal ultrasound showed an endometrial thickness >8 mm [18].

### Hormone assays

Blood samples for FSH, estradiol and leptin measurements were collected between 8:00 and 10:00 am. The first collection occurred on the day pituitary suppression was confirmed for both groups. For patients of group 1, the second blood collection was performed on the last day of controlled ovarian stimulation, i.e., when patients presented the maximum serum levels of estradiol. For patients of group 2, the second blood collection was performed on the day of the insemination (IVF or ICSI), i.e., when they had maximum serum levels of estradiol, and before progesterone administration.

For FSH measurements we used immunometric assays based on enhanced luminescence (AmerliteFSH assay – Amersham International plc – UK). The inter- and intra-assay coefficients of variation were 7.5 and 6.0% respectively. The results were expressed as IU/l. Estradiol was measured using a competitive immunoassay based on enhanced luminescence (AmerliteEstradion-60 assay; Amersham International). The results were expressed as pg/ml. The inter- and intra-assay coefficients of variation were 9.1 and 8.0% respectively.

Leptin was measured in all serum samples in duplicate using a radioimmunoassay method and all samples were assayed in the same batch. The kits (Linco Research – St Charles – USA) contained human leptin antibody prepared in rabbit and raised against highly purified human leptin and standards and tracer prepared with human leptin. The results were expressed as ng/ml. The inter- and intra-assay coefficients of variation were 6.2 and 7.1% respectively.

### Statistics

Shapiro-Wilk test was used to assess the normality of continuous data. The Chi-square test was applied to compare categorical variables between the two groups of patients. Mann-Whitney’s test was performed to compare the medians of BMI, estradiol, leptin, and leptin rate. The Pearson correlation test was used to assess the relationship between the hormones. We calculated the leptin rate (LR) as follows: LR = LA/LB x100, where LA corresponds to the leptin levels after treatment and LB to the leptin levels before treatment. Differences were considered significant when p < 0.05. Descriptive data were expressed as mean ± SD or as median (interquartile range) for normally and non-normally distributed data, respectively.

## Results

All 84 patients selected in this study completed the treatment and had no adverse effects. The mean age (±SD) of the patients submitted to IVF/ICSI was 34.3 ± 3.7 years (range 27 – 42) and for the patients submitted to OD was 42 ± 4.8 years (range 31 – 50). In the GnRHa/FSH group the mean BMI was 23 ± 5.1 and 11 patients (17.2%) were obese, in the GnRHa/E_2_ group the mean BMI was 23.3 ± 4.1 and 3 patients (15%) were obese (Table 1).

**Table 1:** Patient characteristics according to the type of hormonal treatment

	**GnRHa/FSH** **n = 64**	**GnRHa/E**_**2**_ **n = 20**	**p**
Age (years)	34.3 ± 3.7 (27 – 42)	42 ± 4.8 (31 – 50)	0.0001^a^
BMI (Kg/m^2^)	23 ± 5.1	23.3 ± 4.1	0.25 ^a^
Obese	17.2%	15%	0.24 ^b^

Values are expressed in mean ± sd (range).

^a^Student’s *t* test for paired samples.

^b^chi-square test.

When we compared the serum leptin levels according to the BMI, we observed that leptin levels were significantly higher in obese than in non-obese patients, both before (10.9 ± 3.3 and 8.8 ± 4.2, respectively – p = 0.017) and after treatment (26.3 ± 11.7 and 13.5 ± 7.5, respectively – p = 0.0047).

In both GnRHa/FSH and GnRHa/E_2_groups, both serum estradiol and leptin levels were significantly higher after than before starting treatment (Table 2). Leptin levels presented by these groups were similar, both before and after treatment (p = 0.8 and 0.7, respectively). Estradiol levels measured after treatment were significantly higher in the GnRHa/FSH group than in the GnRHa/E_2_ group (p = 0.009) (Table 1).

**Table 2:** Serum Leptin and estradiol levels measured before and after treatment

	**Leptin before treatment**	**Leptin after treatment**	**p**	**E**_**2 **_**before treatment**	**E**_**2 **_**after treatment**	**p**
GnRHa/FSH	10.4 ± 6.8	15.7 ± 9.6	0.0001	16.8 ± 7.8	1440.4 ± 1122.6	0.0001
n = 64	(2.1 - 45)	(4.6 – 46.2)		(9 – 29)	(200–5150)	
GnRHa/E_2_	11.1 ± 9,9	14.5 ± 12.51	0.0079	13.0 ± 6,6	937.9 ± 552.7	0.0001
n = 20	(2.3 – 40.4)	(3 – 53.8)		(9–29)	(108 – 2306)	

Values are expressed as mean ± sd (range).

Data were analyzed using Student’s *t* test for paired samples.

Pearson’s correlation analysis demonstrated significant correlation between FSH or estradiol, and leptin serum levels. Correlation coefficients of 0.72 and 0.92 were observed for the serum levels of leptin detected before and after treatment in the GnRHa/FSHgroup and in the GnRHa/E_2_ group, respectively. Also, this result shows that the final levels of leptin are directly related to the initial ones.

The leptin rate was calculated to evaluate the rising rate of leptin levels from the first to the second analysis. We detected similar leptin rates between obese and non-obese patients (167.4 ± 93.4 and 158 ± 48.9, respectively). The mean leptin rate in the GnRHa/FSH group was 159.6 ± 58.1 (range 74.2 – 408.8) and 136.7 ± 34.2 (range 65.4 – 213.7) in the GnRHa/E_2_ group. The difference between these groups was statistically significant (p = 0.0034), demonstrating a higher rate of increase in leptin levels in the GnRHa/FSH group.

## Discussion

Evidences have suggested a relationship between leptin and the hormones of hypothalamic – pituitary- ovarian axis [9,19-21]. In this study, we analyzed the relationship between leptin and estradiol and FSH, in the absence of interferences of other pituitary and ovarian hormones, due to suppression of pituitary function after administration of GnRHa.

Patients selected in this study were submitted to controlled ovarian hyperstimulation for in vitro fertilization treatment (GnRHa/FSH group) or to oocyte donation treatment (GnRHa/E_2_group). As expected, patients included in the GnRHa/E_2_ group had had higher mean age. These patients were enrolled in a program of egg donation due to the history of low or no ovarian response, usually associated with the increase of age.

Regarding obesity, the two groups showed the same proportion of obese patients, thus excluding bias for comparison between them. When we evaluated the variation in leptin levels according to BMI, the results showed that obese patients had serum leptin levels significantly higher than those of non-obese, both before and after treatment. These results are in accordance to those reported previously [22]. Although the mean age of both groups was different, it has been previously demonstrated that there is no difference in levels of serum leptin between young and postmenopausal women [23].

Pre-treatment and post-treatment evaluations showed that serum levels of estradiol significantly increased in both groups after treatment. These results were expected, since patients presented hypoestrogenism before treatment due to the use of GnRH analogue and after were submitted to controlled ovarian stimulation or to the use of estradiol for endometrial preparation. When comparing the serum levels of estradiol detected after treatment in the two groups, we observed significantly higher levels in the GnRHa/FSH group, due to the response to the ovarian stimulation. In the GnRHa/E_2_ group, patients received fixed doses of estradiol valerate, thus the levels of estradiol detected in serum reach a threshold corresponding to the administered dose.

It is not a consensus whether estradiol interfere with leptin levels. Some studies show that estradiol increases leptin levels in women with normal menstrual cycles [24]. In another study it is shown that estrogen therapies in postmenopausal subjects do not influence serum leptin concentrations [25], and in patients with PCOS, ethinyl estradiol and drospirenone do not increase leptin levels for a 3-month period [26].

With regard to the evaluation of leptin, we also observed a significant increase in its serum levels after treatment, in both groups. These findings demonstrate, for the first time, an elevation of serum leptin levels secondary to the increase in estradiol, in the absence of influence of any other ovarian or pituitary hormone (GnRH dependent), suggesting a direct correlation between these hormones and a role of estradiol in stimulating the production or the release of leptin. We hypothesize that an increase in ob gene (responsible for leptin synthesis by adipocytes) expression induced by estradiol could be the responsible for these results. Another possible mechanism is a leptin gene promoter activation through selective regulation via estrogen receptors [27].

Similar results have already been obtained with women submitted to controlled ovarian stimulation for IVF without the use of GnRHa [20,28,29]. However, differently from our work in which women had pituitary suppression, patients included in these studies were subjected to the influence of ovarian and pituitary hormones, which could interfere with or bias the results.

Although serum levels of leptin were similar in both groups after treatment, the leptin rate was significantly higher in the group of patients treated with rFSH when compared to the group treated with estradiol. This result can be explained by the higher levels of estradiol observed in this group after treatment. However, we cannot exclude the possibility of a direct action of FSH on the production of leptin, through a possible ovarian production or an indirect interference in the synthesis of this hormone by adipocytes, through other ovarian substances [30].

This issue is, however, subject of controversy. On the one hand, it has been shown that leptin levels of women in menopause are lower than those of women in menacme [31]. As in menopause endogenous levels of FSH are very high, these data raise doubt as to whether FSH indeed stimulates the production of leptin. On the other hand, other authors [11,30,32] showed the presence of leptin receptor in ovarian follicles, and the expression of leptin mRNA in granulosa and cumulus oophorus cells. It is possible that the influence of FSH on the ovarian production of leptin occurs only in the presence of follicles, thus not occurring in menopause.

## Conclusions

This study demonstrates a strong correlation between serum levels of estradiol and leptin, suggesting that estradiol is an important regulator of leptin production. These results may contribute to a better understanding of the interaction between metabolic processes and phenomena involved in reproduction. Further investigation is required to establish the role of FSH on the raising of leptin levels.

## Competing interests

The authors declare that they have no competing interests

## Authors’ contributions

SG and MS contributed to the conception and study design, collected the data, performed the interpretation of the data and wrote and approved the final version of the manuscript. AHFB contributed to the interpretation of the data and in the writing of the manuscript. All authors read and approved the final manuscript.
